# Phenotypic and functional consequences of different isolation protocols on skin mononuclear phagocytes

**DOI:** 10.1189/jlb.4A1116-496R

**Published:** 2017-03-07

**Authors:** Rachel A. Botting, Kirstie M. Bertram, Heeva Baharlou, Kerrie J. Sandgren, James Fletcher, Jake W. Rhodes, Hafsa Rana, Toby M. Plasto, Xin Maggie Wang, Jake J. K. Lim, Laith Barnouti, Mark P. Kohout, Tim Papadopoulos, Steve Merten, Norman Olbourne, Anthony L. Cunningham, Muzlifah Haniffa, Andrew N. Harman

**Affiliations:** * Centre for Virus Research, The Westmead Institute for Medical Research, Westmead, New South Wales, Australia;; † The University of Sydney, Sydney, New South Wales, Australia;; ‡ Institute of Cellular Medicine, Newcastle University, Newcastle upon Tyne, United Kingdom;; § Parramatta, New South Wales, Australia;; ¶ Australia Plastic Surgery, Sydney, New South Wales, Australia;; ‖ Cosmetic Culture, Pyrmont, New South Wales, Australia;; # Pure Aesthetics Plastic Surgery, Sydney, New South Wales, Australia;; ** Palm Beach, New South Wales, Australia; and; †† Department of Dermatology, Royal Victoria Infirmary, Newcastle Hospitals NHS Foundation Trust, Newcastle upon Tyne, United Kingdom

**Keywords:** human, DC, macrophage, ex vivo

## Abstract

Mononuclear phagocytes are present in skin and mucosa and represent one of the first lines of defense against invading pathogens, which they detect via an array of pathogen-binding receptors expressed on their surface. However, their extraction from tissue is difficult, and the isolation technique used has functional consequences on the cells obtained. Here, we compare mononuclear phagocytes isolated from human skin using either enzymatic digestion or spontaneous migration. Cells isolated via enzymatic digestion are in an immature state, and all subsets are easily defined. However, cells isolated by spontaneous migration are in a mature state, and CD141 cross-presenting DCs (cDC1) are more difficult to define. Different pathogen-binding receptors are susceptible to cleavage by blends of collagenase, demonstrating that great care must be taken in choosing the correct enzyme blend to digest tissue if carrying out pathogen-interaction assays. Finally, we have optimized mononuclear phagocyte culture conditions to enhance their survival after liberation from the tissue.

## Abstract (precis)

Comparison of skin mononuclear phagocyte isolation techniques on function and subset definition, and the effects of collagenase blends on pathogen binding receptor cleavage.

## Introduction

DCs and macrophages are APCs found in human skin, which form part of the mononuclear phagocyte system [1]. These cells act as immune sentinels performing important, innate functions, as well as bridging adaptive immunity through activation of T cells. DCs and macrophages vary in their ability to phagocytose, but the discerning properties of DCs are their ability to migrate via lymphatics to draining lymph nodes and potent activation of naive T cells, in contrast to macrophages, which are tissue resident. The accessibility of human skin has made it an exemplar tissue to study in vivo mononuclear phagocytes. The early observation of spontaneous migration of DCs and lymphocytes from human skin explants cultured ex vivo provided a reliable experimental tool to isolate these cells [2]. That model was thought to simulate DC activation and lymph node migration from peripheral tissue. However, spontaneous migration ex vivo from skin explants does not necessarily occur through skin lymphatics [3, 4]. Furthermore, fixed, resident macrophages do not “crawl out” but are retained in skin explants [5, 6].

Tissue dissociation enables all mononuclear phagocyte populations to be isolated. Enzymatic digestion is more effective to dissociate skin compared with mechanical disruption because of the resilient nature of the extracellular matrix of human skin. However, caution must be exercised because of the destructive nature of enzymes to surface proteins and carbohydrate moieties. For example, trypsin cleaves many DC surface receptors, including the HIV entry receptor CD4 [7–9], rendering the cells resistant to infection. Thus, pathogen-binding studies using cells isolated via enzymatic digestion must be evaluated in the context of enzyme effects on recognition receptors. In addition, the various isolation protocols and enzymes used also affect maturation status and phenotype with important consequences for isolation purity and for the function of skin mononuclear phagocytes [10–12].

In this study, we evaluated the consequences of existing isolation methods and digestion enzymes on the viability, phenotype, and maturation status of skin mononuclear phagocytes. We present optimized methods for isolating immature cells via enzymatic digestion and discuss the effects of trypsin and 3 commonly used blends of collagenase on cell surface and pathogen-receptor expression. We also highlight the effect of the phenotypic changes during different isolation protocols, particularly in the context of identifying dermal cDC1.

## MATERIALS AND METHODS

### Sources of tissues and ethical approval

This study was approved by the Western Sydney Local Area Health District HREC (reference number HREC/2013/8/4.4[3777] AU RED HREC/13/WMEAD/232). Healthy, human abdominal tissue was obtained from a range of plastic surgeons and written consent was obtained from all donors.

### Tissue processing

Skin was processed by either enzymatic digestion or after spontaneous migration. For both methods, skin was stretched out and sectioned using a skin graft knife (Swann-Morton, Sheffield, United Kingdom), and the resulting skin grafts were passed through a skin graft mesher (Zimmer Biomet, Warsaw, IN, USA) as described by Bond et al. [13]. The meshed skin was placed in RPMI-1640 (Thermo Fisher Scientific, Waltham, MA, USA) with 1U/ml dispase (F. Hoffmann-La Roche, Basel, Switzerland) and 50 µg/ml gentamicin (Thermo Fisher Scientific) and rotated at 4°C overnight. The skin was then washed in PBS and split into dermis and epidermis using fine forceps. To isolate cells via spontaneous migration, the tissue was then cultured for 48 h in media containing 10% FCS, 50 U/ml DNase I (F. Hoffmann-La Roche), and 25 µg/ml gentamicin. For enzymatic digestion, dermal tissue was cut into 25-mm^2^ pieces using a scalpel. Dermal and epidermal tissue was then incubated separately in media containing 100 U/ml DNase I and either 0.5% (w/v) trypsin or 200 U/ml collagenase purchased from either Sigma-Aldrich (Blend F; St. Louis, MO, USA) or Worthington Industries (type IV or type II; Columbus, OH, USA) at 37°C for 30–120 min in a rotator. The cells were then separated from undigested, dermal and epidermal tissue using a tea strainer. For both methods of cell isolation the supernatants were then passed through a 100-μm cell strainer (Greiner Bio-One, Monroe, NC, USA), and the cells were pelleted. The cell pellet was then passed again through a 100-μm cell strainer and was incubated in MACS wash (PBS with 1% human AB serum and 2 mM EDTA) supplemented with 50 U/ml DNase for 15 min at 37°C. The epidermal suspension was spun on a Ficoll-Paque PLUS (GE Healthcare Life Sciences, Little Chalfont, United Kingdom) gradient, and the immune cells were harvested. In some cases, the dermal cells were enriched for CD45-expressing cells using CD45 magnetic bead separation (Miltenyi Biotec, San Diego, CA, USA). Cell suspensions were then counted and/or labeled for flow cytometric phenotyping of surface expression markers or for flow sorting.

### Preparation of in vitro MDDCs and macrophages

CD14^+^ monocytes were cultured with IL-4 and GM-CSF (500 IU/ml and 300 IU/ml, respectively) to produce in vitro–derived MDDCs or were cultured in human serum to generate in vitro–derived, MDM, as described previously [14–18]. Mature MDDCs were prepared by culturing MDDCs with a maturation cocktail (10 ng/ml IL-1β, 1000 U/ml IL-6, 10 ng/ml TNF-α, and 1 µg/ml PGE2) for 48 h.

### Flow cytometry and sorting

Cells were labeled in aliquots of 1 × 10^6^ cells per 100 μl of buffer, according to standard protocols. Nonviable cells were excluded by staining with Live/Dead Near-IR Dead Cell Stain Kit (Thermo Fisher Scientific). Flow cytometry was performed on Becton Dickinson (Franklin Lakes, NJ, USA) LSRFortessa flow cytometer, and data was analyzed with FlowJo software (Tree Star, Ashland, OR, USA). FACS was performed on a Becton Dickinson FACSAria (100 mm nozzle and 137.9 kPa). Sorted cells were collected into FACS tubes containing RPMI-1640 with 10% human AB serum. The Abs were purchased from Becton Dickinson, Miltenyi Biotech, BioLegend (San Diego, CA, USA), Beckman Coulter (Brea, CA, USA), eBioscience (San Diego, CA, USA), and R&D Systems (Minneapolis, MN, USA) as follows: Becton Dickinson: CD45 BV786 (HI30), HLA-DR, BUV395 (G46-6), CD1a BV510 (HI149), CD14 BUV737 (M5E2), Siglec-3 APC (WM53), CD69 APC (L78), CD141 BV711 (1A4), Clec5B APC (DX12), CXCR4 PE (12G5), CD4 APC (RPA-T4), CCR5 PE (2D7), DC-SIGN APC (DCN46), CD80 PE (L307.4), CD83 APC (HB15e), CD86 APC (2331 (FUN-1), Clec12A AF647 (50C1), EpCAM APC (EBA-1), MR APC–BV510 (19.2), mouse IgG1 APC, and mouse IgG1 PE; Miltenyi Biotech: CD14 vioblue (TUK4), Clec7A PE (REA515), Clec9A PE (8F9), CD1c PE-Vio770 (AD5-8E7), CD141 APC (AD4-14H12), Siglec-5 APC (1A5), and langerin vioblue (MB22-9F5); BioLegend: Siglec-1 PE (7-239), and DEC205 PE (HD30); Beckman Coulter: langerin PE (DCGM4) and MR PE (3.29B1.10); eBioscience: CD91 eFluor660 (A2MR-a2); and R&D Systems: L-SIGN PE (120604), DCIR PE (216110), Clec4D PE (413512), Clec4G APC (845404), Clec5A APC (283834), Clec5C APC (239127), Clec6A APC (545943), Clec10A PE (744812), Clec14A APC (743940), Siglec-6 APC (767329), Siglec-9 APC (191240), and Siglec-16 APC (706022).

### Culturing of ex vivo mononuclear phagocytes

Mononuclear phagocytes were cultured for 96 h in the DC culture media as previously described [19], with the following modifications: human AB serum vs. FCS, supplemented with IL-4 and/or GM-CSF, and/or supplemented with 10% or 20% dermal fibroblast conditioned medium was used. Dermal fibroblast condition medium was generated by culturing explanted dermal fibroblasts until 90% confluent in DMEM supplemented with 10% FCS. DMEM was then replaced with DC culture medium, and fibroblasts were cultured for a further 24 h before collection of the medium. Conditioned medium was filtered with a 0.4-µm filter, then stored at −80°C.

### Statistical analysis

All statistical analyses were performed using Prism 6.0 (GraphPad Software, La Jolla, CA, USA). All *P* values are 2-tailed using the Mann-Whitney *U* test.

## RESULTS

### Optimization of enzymatic digestion protocols

**Figure 1.**
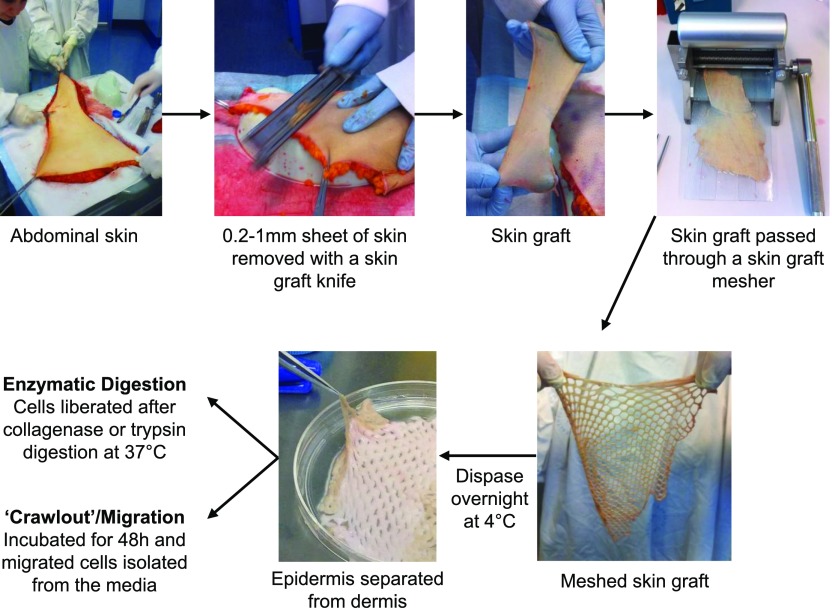
Isolation of mononuclear phagocytes from human skin. Discarded human skin obtained from abdominoplasty operations was processed within 3 h of removal. Skin was stretched, and 0.2–1-mm sheets of skin (skin grafts) were removed with a skin graft knife. Skin grafts were put through a skin graft mesher and then digested with dispase overnight at 4°C. The epidermis was mechanically separated from the dermis, and each was processed separately. Cells were either isolated by digestion with collagenase or trypsin at 37°C (enzymatic digestion) or were allowed to spontaneously migrate from the tissue for 48 h at 37°C.

**Figure 2.**
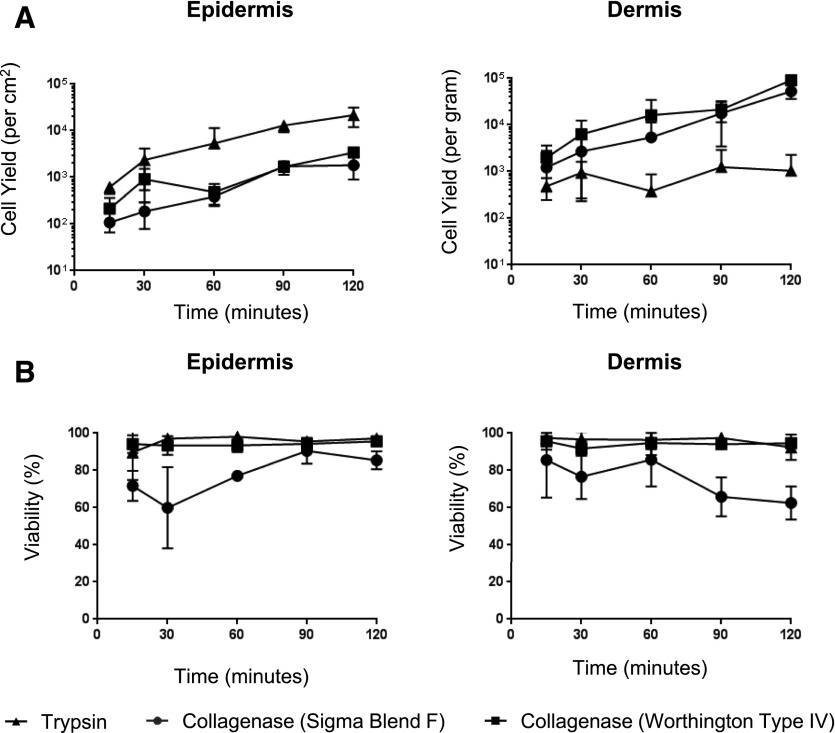
Identification of the optimal enzyme for the isolation of mononuclear phagocytes from skin. Epidermal and dermal tissue was processed as shown in Fig. 1. Each tissue sample was split into roughly 4 equal parts and weighed before digestion with 0.5% trypsin or 200 U collagenase (Worthington type II or IV or Sigma blend F) at 37°C; 1 ml of cell suspension was harvested at 10–30-min intervals for 120 min, and true count beads were added before staining with Live/Dead NIR, HLA-DR PerCP, and CD45 PE.Cy7, and cell suspension was analyzed by flow cytometry for cell yield (A) and viability (B). *n* = 3. The data obtained using type II collagenase was similar to the data for blend F. All enzymatic digestion comparisons were performed on tissue from the same donor.

We optimized an enzymatic digestion protocol using meshed skin grafts to allow for maximal enzymatic access to tissue, followed by overnight treatment with Dispase II at 4°C and then rapid digestion with collagenase blends at 37°C (**Fig. 1**). We used this method to compare the yield and viability of mononuclear phagocytes (CD45^+^, HLA-DR^+^, CD3^−^, CD19^−^) derived from both epidermal and dermal sheets, using either trypsin or 3 commonly used blends of collagenase (types II and IV and blend F) (**Fig. 2**). For the epidermis, we found that trypsin gave the highest cell yield and viability, whereas digestion with both batches of collagenase resulted in much lower yields and, in the case of blend F, poor viability (Fig. 2A). Conversely, for the dermis, we found that trypsin was inferior to collagenase, which liberated good cell yields, but, similar to the epidermis, cells liberated using blend F collagenase had poorer cell viability (Fig. 2B). In all regards, type II collagenase gave results similar to blend F (data not shown).

### Identification of skin mononuclear phagocytes liberated via enzymatic digestion or spontaneous migration

**Figure 3.**
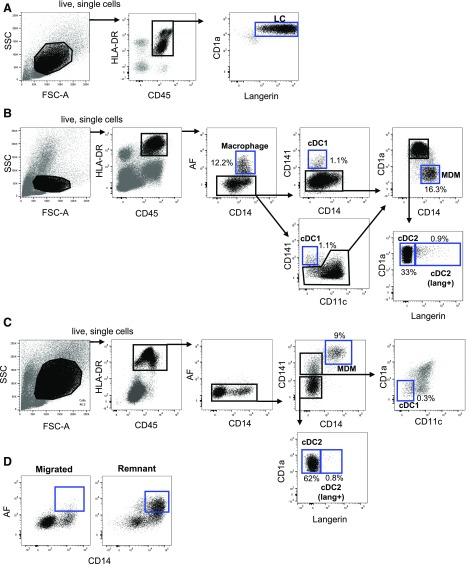
Identification of mononuclear phagocytes from human skin by multiparameter flow cytometry. Cells were isolated from skin with either collagenase digestion (A and B) or spontaneous migration (C). Cells were stained with Live/Dead NIR, HLA-DR BUV395, CD45 BV786, CD1a BV510, CD3 AF700, CD19 BV605, CD11c PE.CF594, CD14 BUV737, CD141 BV711, and langerin vioblue. All epidermal and dermal cells were gated within the live, single HLA-DR^+^CD45^+^CD3^−^CD19^−^ population. (A) Langerhans cells were defined as the CD1a^+^ langerin^+^ cells in the epidermis. (B) Dermal mononuclear phagocytes were gated in sequential order. Macrophages were defined as the autofluorescent CD14^+^ cells. Within the nonautofluorescent population, cDC1s were defined as the CD141^+^CD14^−^ or CD141^+^CD11c^low^ population. The CD141^−^ population was split into CD14^+^ CD1a^−^ cells, MDMs, and 2 populations of CD1a^+^CD14^−^ cDC2s, that could be distinguished by langerin expression. (C) Three populations of nonautofluorescent, dermal, migratory cells could be distinguished; CD14^+^ MDMs were defined as CD141^high^ CD14^+^. The CD141^low^ population was gated on the 2 populations of cDC2s. The CD141^+^CD14^−^ population was gated, and cDC1s were then defined as CD11c^−^CD1a^−^ cells within that gate. (D) CD14^+^ autofluorescent macrophages were present in negligible amounts in the migrated populations but were able to be liberated from tissue by collagenase digestion after 48 h of culture. Representative flow cytometry data are shown, and the relative proportion of each cell subset from the HLA-DR^+^CD45^+^ gate is shown. (A and B) *n* = 50, (C) *n* = 15, (D) *n* = 4.

Skin mononuclear phagocytes share the expression of key surface markers, which can often lead to misidentification of populations [20, 21]. Using flow cytometry analysis of collagenase-liberated cells, we identified epidermal Langerhans cells within the CD45^+^ HLA-DR^+^ fraction (**Fig. 3A**) and the 5 dermal mononuclear phagocyte subsets previously described (Fig. 3B), dermal autofluorescent macrophages [5] and MDMs [3] within the CD14^+^ fraction, cDC1s [22], and cDC2 within the CD14^−^ fraction. A small proportion of dermal cDC2 also expressed langerin, as previously described [23]. Using the spontaneous migration method, we could also identify all dermal cell mononuclear phagocyte populations, with the exception of autofluorescent macrophages (present in negligible amounts, as previously reported by others [3]) (Fig. 3C), and enzymatic digestion of skin explants after 48 h of culture revealed the persistence of these cells within the skin (Fig. 3D).

### Difficulties in identifying cDC1 using spontaneous migration

**Figure 4.**
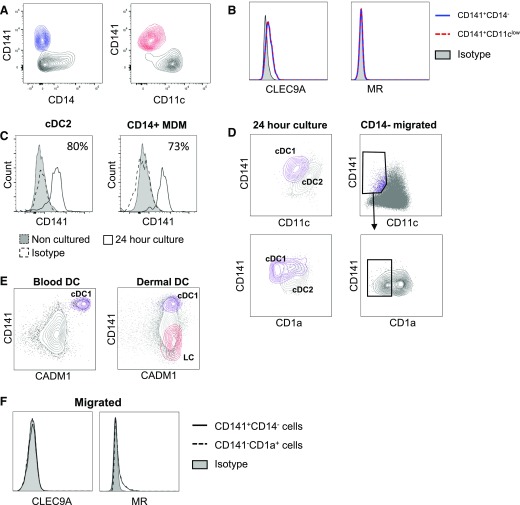
Identifying cDC1 in collagenase digested and spontaneous migrating populations. Cells were isolated by collagenase digestion or spontaneous migration and stained as descried in Fig. 3. After enzymatic digestion cDC1s could be identified by gating on either the CD141^+^CD14^−^ (A) or the CD141^+^CD11c^−^ population (B); both of which expressed CLEC9A but not MR. (C and D) Sorted cDC2s and CD14^+^ MDMs isolated via enzymatic digestion were cultured for 24 h then stained for CD141^+^ (C) and compared with levels from the freshly sorted populations and CD11c and CD1a (D). Gating of CD141 DCs based on CD14 expression (CD141^+^CD14^−^) was compared with gating based on CD11c expression (CD141^+^CD11c^low^). (E) CADM1 expression on cDC1 (purple) compared with all other blood or dermal DC (gray). (F) CLEC9A and MR surface expression of cDC1 and cDC2 in migrated populations.

The correct identification of tissue cDC1 has led to much confusion in the literature [10–12, 24]. Therefore, we next compared the surface marker expression on cDC1s isolated by our enzymatic digestion protocol to spontaneously migrated cells from skin explants (**Fig. 4**). Using collagenase digestion, cDC1s were the only cell population to express CD141, and they could also be identified by their low CD11c expression and/or lack of CD14 expression (Fig. 4A). We further confirmed cells identified using both gating strategies as cDC1 by their coexpression of the cDC1 marker CLEC9A and absence of MR, which is expressed by both cDC2 and CD14^+^ macrophages [25, 26] (Fig. 4B). However, identifying these cells after spontaneous migration was far more difficult because we found high expression of CD141 on CD14^+^ MDMs obtained by spontaneous migration (Fig. 3C). We also found that CD141 was expressed by 86% and 73% of FACS-purified cDC2s and CD14^+^ MDMs, respectively, which had been cultured for 24 h after enzymatic digestion (Fig. 4C). Furthermore, most cDC1s up-regulated CD11c because of spontaneous migration (Fig. 4D, right panel), and after culturing FACS-sorted cDC1 and cDC2 for 24 h after enzymatic digestion, we observed that cDC1s up-regulated CD11c expression such that it was only marginally less than that of cDC2. Furthermore, CD1a expression on sorted cDC1s remained less than that found on cDC2s (Fig. 4D, left panel). CADM1 has recently been shown to reliably identify blood and tissue cDC1s in both mice and humans [27]. However, we found that, although it is a reliable marker to distinguish blood cDC1, it is expressed at similar levels by all skin dermal mononuclear phagocyte populations and is, therefore, not suitable for identifying dermal cDC1s (Fig. 4E). Therefore, using spontaneous migration, we could only identify a tiny fraction of cDC1s via high expression of CD141 and low expression of CD11c and CD1a. Furthermore, CLEC9A and MR expression could no longer distinguish cDC1s from cDC2s because both were rapidly down-regulated on cells that migrated out of the tissue [17, 28] (Fig. 4F).

### Mononuclear phagocytes isolated by spontaneous migration are phenotypically mature

**Figure 5.**
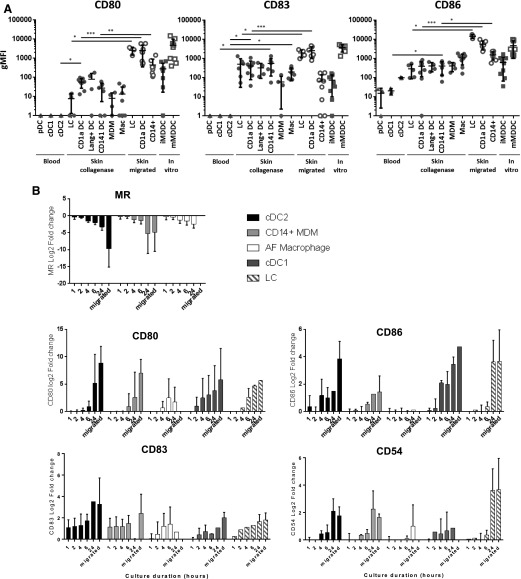
Investigating the maturation phenotype of mononuclear phagocytes derived from collagenase digestion and spontaneous migration. (A) Blood DCs were compared with skin mononuclear phagocytes isolated by either collagenase digestion or spontaneous migration for their expression of CD80, CD83, and CD86 by flow cytometry. Primary mononuclear phagocytes (blood and skin) were also compared with immature and mature MDDCs (iMDDCs and mMDDCs, respectively). The geometric mean fluorescence intensity (gMFI) for each surface expression minus the isotype plotted with the means ± 95% confidence interval. Statistics were generated with the Mann-Whitney *U* test. *P < 0.05; **P < 0.01; ***P < 0.001. Statistics are not shown for the comparison between mMDDCs and all other subsets because they were significantly different to all cells, excluding those isolated by spontaneous migration. (B) Cells isolated from collagenase-digested dermis were cultured in RPMI-1640 supplemented with gentamicin at 37°C for up to 24 h. Cells were collected immediately after digestion and at time intervals up to 24 h and were examined for their expression of CD80, CD83, CD86, CD54, and MR by flow cytometry. Displayed are the means ± sd of the expression at each time point relative to time 0. AF, autofluorescent.

We next determined the maturation status of all human-skin mononuclear phagocytes isolated by digestion and spontaneous migration with peripheral blood DCs and in vitro MDDCs (**Fig. 5A**). We assessed the surface expression levels of the costimulatory molecules CD80, CD83, and CD86, which are up-regulated upon maturation. No blood DCs expressed any CD80 or CD83 and expressed lesser amounts of CD86 compared with skin mononuclear phagocytes. Expression of these markers was consistently greater on skin-derived cells, but cells isolated by spontaneous migration consistently expressed greater amounts of CD80, CD83, and CD86 compared with enzymatic digestion (Fig. 5A). We next determined whether mononuclear phagocytes isolated by our digestion protocol remained in an immature state by determining the maturation marker surface expression levels at regular intervals for 24 h after isolation from tissue (Fig. 5B). CD80, CD83, CD86, and CD54 were progressively up-regulated with time by all tissue mononuclear phagocytes, which began as early as 1 h after extraction in the case of CD83. Even after 24 h in culture, in most cases, collagenase-treated cells did not reach the same level of maturation that cells derived by spontaneous migration reached. We also observed down-regulation of the CLR MR, which, similar to other pathogen-binding CLRs, is down-regulated upon maturation [17, 28].

### Effect of collagenase and skin culture on expression of surface markers and pathogen-binding receptors

When isolating cells by enzymatic digestion, it is important to know whether the enzymes used can cleave the cell surface receptors, especially for CLRs if pathogen-binding studies are being performed. We examined the effect of collagenase and trypsin on surface molecules used to define DC subsets (CD1a, CD1c, CD11c, CD14, CD45, CD141, CD207/langerin, and HLA-DR) as well as HIV entry receptors (CD4, CCR5, and CXCR4), maturation markers (CD80, CD83, and CD86), and a large panel of lectins, including CLRs and Siglec (**Table 1**). In each case, we used a DC subset that is known to express those markers and compared the expression levels using type IV and blend F collagenase. In cases in which we could not detect expression on cells treated with either enzyme, we confirmed the absence of mRNA (data not shown), suggesting that the lack of detection was not due to complete enzyme cleavage.

**TABLE 1.** Collagenase cleavage of surface molecules

Marker	Subset tested	Blend F (gMFI)		Type IV (gMFI)		Trypsin (gMFI)		Statistics*^a^*	Best enzyme
		Means	sd	Means	sd	Means	sd		
Population markers									
HLA-DR	LC	6094	2128	4975	2947	3259	894	NS	NA
CD45	LC	7544	4211	12,974	7724	4799	2629	NS	NA
CD1a	LC	41,052	11,087	38,823	10,006	5638	1395	NS	NA
CD1c	CD1a	2193	108	8771	5543	1063	213	**	Type IV
CD11c	CD1a	2118	41	3094	218	106	21	NS	NA
CD14	CD14	2254	796	3262	2291	548	24	NS	NA
CD141	CD141	3534	2213	2403	1239	245	83	NS	NA
Langerin	LC	4753	2915	20262	18157	7516	5349	*	Type IV
Epcam	LC	21,227	15,762	8448	9146	10,768	1249	NS	NA
Maturation markers									
CD80	ALL*^b^*	—	—	—	—			—	NA
CD83	CD1a	—	—	522	150			**	Type IV
CD86	CD1a	180	90	406	105			*	Type IV
HIV entry receptors									
CD4	CD1a	322	125	843	257	427	49	*	Type IV
CXCR4	CD14	—	—	156	51	189	97	**	Type IV
CCR5	CD1a	454	323	39	53	203	38	**	Blend F
CLRs
CLEC4A (DCIR)	CD1a	13770	4813	631	142	250	88	**	Blend F
CLEC4D	CD14	575	271	136	123			**	Blend F
CLEC4E	CD14	7021	3008	4890	1840			NS	NA
CLEC4G	ALL	207	31	74	40			**	Blend F
CLEC4L (DC-SIGN)	Mac	1264	450	152	205	0	—	**	Blend F
CLEC4M	CD14	2077	621	165	135			**	Blend F
CLEC5A	CD1a	18,772	7665	16,767	4976	3698	488	NS	NA
CLEC5B	ALL*^b^*	—	—	—	—			—	NA
CLEC5C	ALL*^b^*	—	—	—	—			—	NA
CLEC6A	ALL*^b^*	—	—	—	—			—	NA
CLEC7A	CD1a	508	139	702	137			NS	NA
CLEC8A	CD14	263	99	90	118			*	Blend F
CLEC9A	CD141	186	66	188	116			NS	NA
CLEC10A	CD14	561	334	44	43			*	Blend F
CLEC12A	CD14	5042	869	1439	1211			*	Blend F
CLEC13B (DEC-205)	CD1a	518	321	428	276	2823	488	NS	NA
CLEC13D (MR)	CD14	6571	712	2219	582	0	—	**	Blend F
CLEC14A	CD141	110	105	119	118			NS	NA
Siglecs									
Siglec-1	CD14	1168	578	316	306			*	Blend F
Siglec-3	CD1a	12,616	12,452	5644	4117	1938	190	NS	Blend F
Siglec-5	CD1a	668	126	298	350			NS	Blend F
Siglec-6	ALL*^b^*	—	—	—	—			—	NA
Siglec-9	CD1a	3757	2304	4827	3767			NS	NA
Siglec-16	CD1a	181	175	144	99			NS	NA
Other									
CD69	CD1a	3123	895	2876	660			NS	NA
CD91	CD14	212	43	0	0			**	Blend F

Human skin mononuclear phagocytes were isolated from human skin with either blend F or type IV collagenase, and the surface expression was determined for a range of markers on a specific cell subset by flow cytometry with the gating strategy shown in Fig. 3. For each marker, the cell subset was analyzed, and the MFI of surface marker expression after extraction with each enzyme blend (*n* = 5) was determined. Epcam, epithelial cell adhesion molecule; FC, fold change; gMFI, geometric mean fluorescence intensity; NA, not applicable; NS, not significant. *P ≤ 0.05; **P ≤ 0.01.

a Mann-Whitney *U* test.

b No expression on any subset, confirmed by RNAseq.

Trypsin did not significantly cleave CD45, HLA-DR, EpCAM, CD1a, or langerin, which is consistent with its suitability in isolating Langerhans cells from epidermis. However, it did cleave key dermal DC isolation markers, such as CD11c, CD1c, CD141, and CD14, rendering it unsuitable for the isolation of dermal mononuclear phagocytes. Furthermore, it cleaved many pathogen-binding receptors, including those expressed on Langerhans cells, such CD4, CCR5, DEC205, and DCIR. Blend F collagenase cleaved CD1c, but all other markers used to discriminate mononuclear phagocyte cell subsets were expressed at similar levels, regardless of the collagenase blend used. Blend F also cleaved CD83, CD86, CD4, and CXCR4. In distinct contrast, type IV collagenase cleaved many CLRs and Siglecs as well as CCR5 and CD91. This highlights the importance of choosing the correct enzyme when designing experiments.

### Optimization of DC culture conditions

Maintaining a viable population of skin mononuclear phagocytes in culture is critical for downstream functional assays. Therefore, we compared the consequences of culture conditions on the survival of FACS-purified skin mononuclear phagocyte populations (**Table 2**). We observed no difference in cell viability between cells cultured in human AB serum or FCS, but culture with IL-4, but not GM-CSF, increased the viability of CD14 MDMs. Furthermore, the addition of dermal fibroblast-conditioned medium increased cell viability of all cell populations, with 20% supplementation giving the best results and autofluorescent macrophages showing the most enhanced cell survival.

**TABLE 2.** Ex vivo skin mononuclear phagocyte culture optimization

Parameter	IL−4	GM−CSF	FCM (%)	Fold change in cell viability	% live
cDC2	−	−	−	1.0	23
	+	−	−	0.5	11
	−	+	−	0.9	21
	+	+	−	1.1	25
	−	−	20	1.4	32
	−	−	40	1.1	25
CD14 monocyte–derived macrophages	−	−	−	1.0	13
	+	−	−	3.6	47
	−	+	−	1.6	21
	+	+	−	2.7	35
	−	−	20	3.3	43
	−	−	40	2.2	29
AF^+^ macrophages	−	−	−	1.0	12
	+	−	−	1.0	12
	−	+	−	0.3	3.6
	+	+	−	1.1	13
	−	−	20	4.9	59
	−	−	40	2.6	31

Human skin mononuclear phagocytes were isolated from human skin with type IV collagenase, were sorted by FACS, and then cultured for 96 h in 96-well, round-bottom plates at 2 × 10^5^ cells/well under various conditions. The percentage of live cells was then calculated using live/dead near-infrared staining and true count beads (*n* = 3). AF, autofluorescence; FCM, fibroblast conditioned media.

## DISCUSSION

Studying human cells at the portals of pathogen entry is important for advancing our understanding of tissue leukocyte responses to microorganisms. This is especially the case when investigating viruses, such as HIV-1 and HSV, in which specific subsets of those cells have been shown to have a direct and critical role in transmission and/or immune clearance [8, 29–31]. The ethical considerations and logistical difficulties of human in vivo studies with pathogens highlights the critical need for a robust and minimally disruptive isolation strategy of leukocytes from human tissues. Different isolation methods affect leukocyte phenotype, activation status, and pathogen-binding receptor expression. Therefore, in this study, we present an optimized protocol for extracting mononuclear phagocytes from tissue in an immature state and flow cytometry gating strategies to accurately define all currently known subsets isolated by either enzymatic digestion or spontaneous migration. We then determined the functional consequences of each method of isolation on maturation status and also compared the ability of different blends of collagenase to cleave a wide range of pathogen-binding receptors. Finally, we optimized the culturing conditions for enhanced mononuclear phagocyte survival after isolation.

Our findings show marked phenotypic differences between mononuclear phagocytes isolated by spontaneous migration from skin explants and enzymatic skin digestion. The consequences of this relate directly to difficulties in isolating specific populations, such as cDC1s. We demonstrate here that cDC1s can be easily defined when liberated from tissue by enzymatic digestion because they are the only mononuclear phagocyte population to express CD141, do not express CD14, and express lower levels of CD11c. However, these cells are much more difficult to confidently define when liberated by spontaneous migration because a large proportion of both CD14^+^ MDMs and cDC2s begin to express CD141, and cDC1s up-regulate CD11c. Furthermore, cDC1s have also been shown to up-regulate the dermal cDC2 marker CD1a [12]. A recent study suggests that CADM1 is uniquely expressed by cDC1 in both mice and humans and could be a reliable marker to define those cells in both tissue and blood [27]. However, we show here that CADM1 is expressed by all human skin mononuclear phagocytes. Taken together, this study shows that, in the absence of a reliable XCR1 Ab, care must be taken in the proper definition of cDC1. Previously, 3 populations identified as dermal cDC1s based on different gating and isolation strategies were revealed to be unrelated by subsequent flow cytometry and transcriptomics meta-analysis [10–12, 24]. Only one study isolated the dermal equivalent of blood cDC1s [12, 24], and it is notable that it was the only study that isolated those cells by enzymatic digestion. The other 2 studies analyzing spontaneously migrated cells highlight the inherent difficulties resulting from promiscuous expression of CD141 on other DCs and endothelial cells [3, 21].

Tissue mononuclear phagocytes capture pathogens via an array of pathogen-binding receptors, such as CLRs and Siglecs [29, 32–35]; however, many of those receptors, such as DC-SIGN and MR, are down-regulated upon maturation [17, 29, 36]. Therefore, it is important to isolate cells in an immature state when investigating their interactions among pathogens. Accordingly, we compared the maturation status of cells isolated by each method. We found that skin mononuclear phagocytes isolated by enzymatic digestion had greater immaturity than did those isolated by spontaneous migration but were more mature that those isolated from blood. We found that cells isolated by enzymatic digestion rapidly began to undergo maturation after extraction and that down-regulation of the pathogen-binding receptor MR began to occur within 1 h. This may be due to exposure to activation signals during the isolation process. It is notable, however, that, in most cases, even after 24 h in culture, enzymatically liberated cells never reached the same level of phenotype maturation as those isolated by spontaneous migration. These findings illustrate that while ex vivo cells can be used for pathogen interaction studies, the experiments must be carried out immediately after isolation.

Importantly, the spontaneous migration method can only be used for DCs, as other leukocytes such as macrophages and lymphocytes do not migrate out the tissue. Of note, spontaneous migration may occur without cells entering cutaneous lymphatic vessels [3, 4]. Indeed, CCR7-deficient peripheral tissue murine DCs are unable to migrate to draining lymph nodes but can still spontaneously migrate from skin explants cultured ex vivo without entering lymphatic vessels [4]. Analysis of these skin explants showed that migrating cells did not form organized chords through lymphatic vessels but were randomly distributed [4].

An important consideration when isolating cells via enzymatic digestion is the vulnerability of cell surface receptors to enzymatic cleavage. The enzymatic dissociation protocol used in this study liberates mononuclear phagocytes in an immature state while still expressing pathogen-binding receptors. A good example of the relevance of such concerns is in studies of HIV transmission, where the essential HIV entry receptor CD4 can be cleaved by trypsin [8]. Furthermore, HIV only infects immature DCs that express the entry coreceptor CCR5, which is rapidly down-regulated upon maturation. In this study, we confirmed that CD4 is cleaved by trypsin, which also cleaves many other surface proteins, including dermal DC identification markers and many pathogen-binding receptors, including those expressed by Langerhans cells. Therefore, although trypsin generates high yields of Langerhans cells, it may be a poor choice for pathogen-interaction studies, especially HIV. Furthermore, we show that collagenase blends markedly differ in the surface proteins they cleave. Surprisingly, although type IV collagenase is specifically designed for reduced cell-surface cleavage, we found that the enzyme dramatically cleaved many CLRs and Siglecs.

Mononuclear phagocyte cell culture after tissue isolation is a major limiting factor in performing functional assays. We found that 20% fibroblast-conditioned medium enhanced the survival of all CD14^+^ cell populations, especially autofluorescent tissue macrophages, and that IL-4, but not GM-CSF, enhanced MDM survival.

Our study demonstrates an enzymatic digestion protocol for the isolation of human skin mononuclear phagocytes in an immature state with minimal surface-receptor cleavage. It demonstrates the need for care in the correct identification of cDC1s, especially when isolated by spontaneous migration. Finally, we highlight the importance of enzyme choice for skin digestion for pathogen-interaction studies.

## AUTHORSHIP

R.A.B. and K.M.B. performed and supervised the bulk of the work in the laboratory of A.N.H. with the aid of H.B., K.J.S., J.W.R., H.R., and T.M.P; J.F. performed the CADM1 staining in the laboratory of M.H; and X.M.W. provided intellectual input into the flow cytometry panel design. J.J.K.L., L.B., M.P.K., T.P., S.M., and N.O. are plastic surgeons and provided tissue and intellectual input into tissue-processing techniques. A.L.C. and M.H. provided significant intellectual input and help with manuscript preparation. A.N.H. conceived the study and prepared the manuscript.

## Abbreviations

CADM1: cell-adhesion molecule 1
cDC1: CD141 cross-preventing DCs
cDC2: CD1a^+^/CD1c^+^ DCs
CLR: C-type lectin receptor
DC: dendritic cell
DCIR: dendritic cell immunoreceptor
DC-SIGN: dendritic cell–specific intercellular adhesion molecule 3 grabbing nonintegrin
HREC: Human Research Ethics Committee
MDDC: monocyte-derived dendritic cell
MDM: monocyte-derived macrophage
MR: mannose receptor
Siglec: sialic acid–binding immunoglobulin-type lectin
